# Measuring depression in Primary Health Care in Spain: Psychometric properties and diagnostic accuracy of HSCL-5 and HSCL-10

**DOI:** 10.3389/fmed.2022.1014340

**Published:** 2023-01-09

**Authors:** María Rodríguez-Barragán, María Isabel Fernández-San-Martín, Ana Clavería, Jean Yves Le Reste, Patrice Nabbe, Emma Motrico, Irene Gómez-Gómez, Eva Peguero-Rodríguez

**Affiliations:** ^1^Primary Health Centre La Mina, Gerència Territorial d’Atenció Primària de Barcelona, Institut Català de la Salut, Sant Adrià de Besòs, Barcelona, Spain; ^2^Institut Universitari d’Investigació en Atenció Primària Jordi Gol (IDIAP Jordi Gol), Barcelona, Spain; ^3^Faculty of Medicine, Department of Pediatrics, Obstetrics, Gynecology and Preventive Medicine, Universitat Autònoma de Barcelona, Barcelona, Spain; ^4^Gerència Territorial d’Atenció Primària de Barcelona, Institut Català de la Salut, Barcelona, Spain; ^5^I-Saúde Group, South Galicia Health Research Institute [IISGS-Servicio Gallego de Salud (SERGAS)], Vigo, Spain; ^6^Vigo Health Area, Servicio Gallego de Salud (SERGAS), Vigo, Spain; ^7^Network for Research on Chronicity, Primary Care and Health Promotion (RICAPPS), Vigo, Spain; ^8^Department of General Practice, ER 7479 SPURBO Soins Primaires, Santé Publique, Registre des Cancers de Bretagne Occidentale, Université de Bretagne Occidentale, Brest, France; ^9^Department of Psychology, Universidad Loyola, Andalucía, Spain; ^10^Primary Health Centre El Castell, Gerència Territorial d’Atenció Primària Metropolitana Sud, Institut Català de la Salut, Castelldefels, Barcelona, Spain; ^11^Departamento de Ciencias Clinicas, Facultad de Medicina, Universidad de Barcelona, Barcelona, Spain

**Keywords:** depression, Hopkins Symptom Checklist, Primary Health Care (MeSH), patient reported outcome measures (MeSH), diagnostic accuracy, psychometric properties

## Abstract

**Background:**

Depression has a high prevalence among European countries. Several instruments have been designed to assess its symptoms in different populations. The Hopkins Symptom Checklist 25 (HSCL-25) scale has been identified as valid, reproducible, effective, and easy to use. There are short versions of this scale that could be useful in Primary Care (PC) settings, but their psychometric properties are unknown.

**Aim:**

To assess in PC patients the psychometric properties and diagnostic accuracy of the Spanish version of the HSCL-10 and the HSCL-5 consisting of 10 and 5 items, respectively.

**Methods:**

A multicenter, cross-sectional study was carried out at six PC centers in Spain. The HSCL-25 was administered to outpatients aged 45–75 who also participated in the structured Composite International Diagnostic Interview (CIDI). HSCL-10 and HSCL-5 were assessed and compared to HSCL-25 regarding total score correlation, internal consistency, and criterion validity against the gold-standard CIDI. This is a methodological study from a secondary data analysis and the primary data has been previously published.

**Results:**

Out of 790 patients, 767 completed the HSCL-25 and 736 the CIDI interview (96.0%). Cronbach’s Alpha was 0.84 for HSCL-10 and 0.77 for HSCL-5. The known-group method and confirmatory factor analysis were acceptable for the establishment of construct validity. Sensitivity was 79.7% (CI95%, 67.7–88.0%) for HSCL-10, and 78.0% (CI95%, 65.9–86.6%) for HSCL-5, whereas specificity was 83% (CI95%, 80.0–85.7%) for HSCL-10, and 72.8% (CI95%, 69.3–76.0%) for HSCL-5. Area under the curve against CIDI was 0.88 (CI95%, 0.84–0.92%) for HSCL-10, and 0.85 (CI95%, 0.81–0.89%) for HSCL-5. Optimum cutoff point calculated with Youden Index was 1.90 for the HSCL-10 and 1.80 for the HSCL-5.

**Conclusion:**

HSCL-10 and HSCL-5 are reliable and valid tools to detect depression symptoms and can be used in PC settings.

## Introduction

Mental health is an issue of increasing concern in Spain and other European countries and represents a considerable percentage of Primary Care (PC) consultations. The clinical diagnosis of mental disorders is based on the symptoms of the Diagnostic and Statistical Manual of Mental Disorders (DSM). In addition, there are many questionnaires available which assess mental health diseases in populations and are frequently employed in PC (1). Such instruments are used for the purposes of screening and symptom detection to aid diagnosis.

The United States Preventive Services Task Force (USPSTF) recommends universal screening for depression in the general adult population (2) as it has been shown to reduce symptoms and improve quality of life and functional status (3). Screening should, however, be combined with adequate systems to ensure accurate diagnosis, effective treatment, and appropriate follow-up.

Up to 75% of patients with depression are treated exclusively in PC (4). Nevertheless, Family Doctors (FD) report difficulties in the identification of mental health disorders (5) and consider that evaluative tools permitting rapid diagnosis with a limited number of items are required (6). The issue of time is particularly relevant within the context of PC given its complexity and limited consultation schedules. Moreover, FD have been found to feel more confident in detecting and managing depression when employing questionnaires (7).

The ideal questionnaire should be reliable, valid, ergonomic, and easy to use for both patients and healthcare professionals. It should be validated according to the Consensus-based Standards for the Selection of Health Measurement Instruments (COSMIN) (8) for the language and population they are directed at.

The Hopkins Symptom Checklist 25 (HSCL-25) (9) is a widely used, self-administered screening tool for depression and psychological distress; it is also employed for research purposes. It has been shown to be valid and reliable for different populations (10–14), useful in PC (15), and it is available in several languages (16) including Spanish. The Spanish version of HSCL-25 was translated, culturally adapted, and validated following the COSMIN (17, 18).

The HSCL-25 is a short version of the Symptom Checklist 90-Revised (SCL-90-R) (19). There are, however, other questionnaires with fewer items (20) which are of interest due to their brevity and reduced completion time. Shorter versions are useful as they can save time in complex settings such us the PC context. The HSCL-10 and HSCL-5 were developed by selecting 10 and 5 items, respectively, due to their strong correlation with the HSCL-25 mean score (19, 21). Items of the HSCL-10 and HSCL-5 are included in the longer HSCL-25. Both the HSCL-10 and the HSCL-5 have shown a strong mean score correlation with respect to the HSCL-25 and high reliability (Cronbach’s Alpha Coefficient >0.80) (22). Both short versions, especially the HSCL-10, have been used in different populations such as adolescents (23–25), industry workers (26), population surveys (22, 27), patients with alcohol use disorder (28), refugees (29), and also to measure symptoms of depression and anxiety due to the COVID pandemic (30–32). Their psychometric properties recommend them for clinical use as screening and symptom assessment tools and for research purposes (25, 27, 33).

The aim of this article is to report the psychometric properties and diagnostic accuracy of the Spanish versions of the HSCL-5 and HSCL-10 for their use as rapid and accessible depression screening instruments in PC.

## Materials and methods

### Data collection, study population, and variables

The present study was based on data from a cross-sectional multicenter study designed to validate the HSCL-25 in a Spanish PC population. This is a methodological study from a secondary data analysis and the primary data has been previously published (18). Participants were patients attending six Spanish Primary Health Centers (PHC) taking part in the EIRA study (34, 35). Inclusion criteria were to be aged 45–75 years and presenting two or more of the following: Smoking, low adherence to the Mediterranean dietary pattern, and insufficient physical activity. Exclusion criteria were: Advanced serious illness, cognitive impairment, dependence in basic everyday activities, severe mental illness, unable to attend the PHC, under treatment for cancer or in end-of-life care, or planning to travel during the intervention period.

Participants were recruited by consecutive sampling of patients attending the PHC for any reason during a 6-month-period in 2017. They were asked to complete sociodemographic data (gender, age, nationality, marital status, current employment, and education level) and the self-administered HSCL-25 questionnaire (and other questionnaires/forms related to the EIRA study). Afterward, trained professionals, blinded to the HSCL-25 score, conducted the gold-standard CIDI interview with all participants.

### Hopkins symptom checklist-25 (HSCL-25)

The HSCL-25 is a widely used, self-administered questionnaire designed to measure anxiety and depression symptoms (9, 11) and takes 5–10 min to complete (13). It consists of 10 and 15 items belonging to the anxiety and depression dimensions, respectively. The items are answered on a four-point Likert-like scale: 1 = “Not at all;” 2 = “A little;” 3 = “Quite a bit;” 4 = “Extremely.” The average score, ranging from 1 to 4, is calculated by dividing the total score by the number of the items. A cutoff value of 1.75 is generally used for major depression diagnosis, as it is considered a valid predictor of mental disorder (10, 13, 36).

Items belonging to HSCL-25, HSCL-10, and HSCL-5 are shown in Table 1. The corresponding cutoffs points are 1.85 for the HSCL-10 and 2.00 for the HSCL-5 (22). The Spanish version of the HSCL-25 was used in this study (17).

**TABLE 1:** Items belonging to HSCL-25, HSCL-10, and HSCL-5 scale.

Item in HSCL-25	HSCL-10	HSCL-5	
1. Being scared for no reason	X		Anxiety dimension
2. Feeling fearful	X	X	
3. Faintness	X		
4. Nervousness		X	
5. Heart racing			
6. Trembling			
7. Feeling tense	X		
8. Headache			
9. Feeling panic			
10. Feeling restless			
11. Feeling low in energy			Depression dimension
12. Blaming oneself	X		
13. Crying easily			
14. Losing sexual interest			
15. Feeling lonely			
16. Feeling hopeless	X	X	
17. Feeling blue	X	X	
18. Thinking of ending one’s life			
19. Feeling trapped			
20. Worrying too much		X	
21. Feeling no interest			
22. Feeling that everything is an effort	X		
23. Worthless feeling	X		
24. Poor appetite			
25. Sleep disturbance	X		

Items belonging to depression and anxiety dimensions.

### Composite international diagnostic interview (CIDI)

The CIDI is a well-known, standardized interview designed by the World Health Organization (WHO) based on the fourth edition of the Diagnostic and Statistical Manual of Mental Disorders (DSM-IV) and the International Classification of Diseases-10 (ICD-10) criteria (37). It is administered by trained interviewers and available in different languages (38). For this study, section E (questions referring to depression) of the Spanish version was used. The CIDI was conducted by trained psychologists.

As the diagnose of depression is a clinical interview performed by a trained professional and conducted using the DSM criteria, the CIDI is considered the gold-standard in the present study.

### Ethical considerations

The study was developed according to national and international legislation (the Declaration of Helsinki and latest versions). The protocol was evaluated by the IDIAP Jordi Gol Ethical Research Committee (approval number: P16/025) and by the corresponding regional governments. Written consent was obtained from the participants, and the questionnaires were codified with an identification number to protect anonymity and confidentiality.

### Statistical analysis

Analysis was conducted with STATA version 15. Missing values for the HSCL-25 were replaced with the individual mean for the rest of the items. Subjects with ≥50% missing items were excluded.

Total score was calculated for HSCL-25, HSCL-10, and HSCL-5 for the total population and in relation to gender and age categories, and by the following sociodemographic groups: marital status, education level, and current employment.

Reliability of the HSCL-10 and HSCL-5 was analyzed by calculating the Cronbach’s Alpha Coefficient and for each of the two depression and anxiety subscales. A value of ≥0.7 was considered adequate (39). Cronbach’s Alpha without the item was also calculated to assess the contribution of each item to the internal consistency of two versions.

Construct validity was measured with the known-groups method by comparing the total score of the HSCL-10 and the HSCL-5 by gender. The total score was expected to be significantly higher among women (40, 41). Independent sample *t*-test was performed, a significant result (*p* < 0.05) was considered satisfactory (42). Confirmatory factor analysis (CFA) was conducted to assess the structural validity. To evaluate the estimated model fit, the absolute fit index was calculated with chi-squared distribution. Given that this value may be affected by the sample size, complementary indices were employed: The root mean square error of approximation (RMSEA), the standardized root mean square residual (SRMR), the comparative fit index (CFI), and the Tucker–Lewis fit index (TLI) (43, 44). Cutoff values considered adequate were: SRMR < 0.05, RMSEA ≤ 0.08, CFI > 0.90, and TLI > 0.90 (45).

Criterion validity and diagnostic accuracy were measured by calculating the ROC curve for the HSCL-10 and HSCL-5 scale in comparison with the gold-standard CIDI (8). The area under the curve (AUC) was estimated with 95% confidence interval (CI95%). Best cutoff points for the study population and by gender were calculated with the Youden Index for both HSCL-10 and HSCL-5. Youden Index is defined as “Sensitivity + Specificity −1,” it is a value that indicates the validity of the instrument for a specific cutoff point (46). Sensitivity and specificity were assessed as measures of internal validity; positive and negative predictive values were also calculated. Both the sensitivity and specificity of a screening test should be greater than 0.70 (42). For these calculations, cutoffs those proposed by Strand et al. were followed: 1.85 for the HSCL-10 and 2.00 for the HSCL-5 (22). Other authors have employed the same cutoff points (47).

## Results

### Participants

From a total of 790 patients, 767 completed the HSCL-25 (97.1% response rate). Participants’ mean age was 58.4 years (± 8.2), 54.4% were women, and there were no significant gender differences among age categories. Table 2 shows the mean score and standard deviation (SD) of the three HSCL versions in relation to the sociodemographic characteristics of the sample.

**TABLE 2:** HSCL-25, HSCL-10, and HSCL-5 scale mean scores and standard deviation (SD) in relation to age, gender, marital status, education, and current employment.

	HSCL-25					HSCL-10			HSCL-5		
	*n*	%	Mean	*SD*	*P*-value	Mean	*SD*	*P*-value	Mean	*SD*	*P*-value
**Total**	767		1.57	0.45		1.55	0.50		1.72	0.59	
**Gender**											
Men	350	45.6	1.42	0.35	<0.001	1.41	0.39	<0.001	1.53	0.46	<0.001
Women	417	54.4	1.69	0.49		1.68	0.55		1.88	0.64	
**Age**											
45–54	298	38.7	1.63	0.47	0.006	1.62	0.52	0.005	1.71	0.61	0.060
55–64	265	34.7	1.55	0.49		1.54	0.51		1.71	0.61	
65–75	204	26.6	1.50	0.40		1.47	0.44		1.64	0.54	
**Marital status**											
Married/with a partner	550	71.9	1.54	0.43	0.028	1.52	0.47	0.029	1.69	0.56	0.084
Single	57	7.3	1.58	0.44		1.58	0.48		1.73	0.59	
7-810-11 Separated or divorced	103	13.3	1.68	0.48		1.69	0.56		1.82	0.67	
Widow (er)	57	7.5	1.64	0.51		1.60	0.61		1.86	0.65	
**Education**											
Primary or lower	398	51.8	1.57	0.42	0.855	1.54	0.48	0.697	1.74	0.58	0.333
Secondary or higher	368	48.2	1.56	0.47		1.56	0.52		1.70	0.60	
**Current employment**											
Employed	313	41.0	1.55	0.43	0.547	1.54	0.48	0.774	1.84	0.63	0.746
Housewife	111	14.5	1.64	0.45		1.61	0.51		1.82	0.60	
Unemployed	83	10.9	1.65	0.46		1.65	0.55		1.82	0.60	
Retired	209	27.3	1.45	0.40		1.43	0.44		1.57	0.54	
Others (student, sick leave, and disability)	48	6.3	1.88	0.53		1.88	0.58		1.98	0.58	

There were statistically significant differences in total scores for the three versions regarding gender. Women scored higher with a minimum difference >0.25 points. There were also statistically significant differences in the total scores of the HSCL-25 and HSCL-10, but not the HSCL-5, with respect to age and marital status. No differences were observed regarding education level or current occupation.

### Reliability: internal consistency

Cronbach’s Alpha was 0.8417 and 0.7712 for the HSCL-10 and HSCL-5, respectively. When analyzing the two dimensions separately, this value was higher for the depression dimension than the anxiety one. These values, and the value of the coefficient without the item, are depicted in Table 3.

**TABLE 3:** Cronbach’s alpha coefficient without the item and for total values.

Item	HSCL-25	HSCL-10	HSCL-5
1. Being scared for no reason	0.9148	0.8359	
2. Feeling fearful	0.9137	0.8307	0.7580
3. Faintness	0.9127	0.8268	
4. Nervousness	0.9109		0.7156
5. Heart racing	0.9136		
6. Trembling	0.9151		
7. Feeling tense	0.9109	0.8205	
8. Headache	0.9163		
9. Feeling panic	0.9156		
10. Feeling restless	0.9117		
11. Feeling low in energy	0.9110		
12. Blaming oneself	0.9131	0.8279	
13. Crying easily	0.9145		
14. Losing sexual interest	0.9153		
15. Feeling lonely	0.9121		
16. Feeling hopeless	0.9127	0.8261	0.7427
17. Feeling blue	0.9085	0.8080	0.6820
18. Thinking of ending one’s life	0.9159		
19. Feeling trapped	0.9129		
20. Worrying too much	0.9129		0.7399
21. Feeling no interest	0.9129		
22. Feeling that everything is an effort	0.9112	0.8188	
23. Worthless feeling	0.9140	0.8315	
24. Poor appetite	0.9167		
25. Sleep disturbance	0.9152	0.8424	
Total	0.9166	0.8417	0.7712
Anxiety subscale (items 1–10)	0.8306	0.6859	0.5404
Depression subscale (items 11–25)	0.8784	0.7804	0.7010

The most consistent item for the HSCL-10 was 17 “Feeling blue” followed by 22 “Feeling that everything is an effort.” The least consistent was 25 “Sleep disturbance” followed by 1 “Being scared for no reason.” Item 25 “Sleep disturbance” had a Cronbach’s Alpha without the item of 0.8424, as a result, this item worsened the reliability of this version, as this value was above 0.8417. In the HSCL-5, item 17 “Feeling blue” was also the most consistent whilst two “Feeling fearful” was the least consistent although without affecting reliability.

### Construct validity: Known-groups method and confirmatory factor analysis (CFA)

The known-group method analysis showed that the total score of the HSCL-10 indicated that women had significantly higher scores (mean = 1.68; SD 0.03) than men (mean = 1.41; SD 0.02; *t* = 7.76; *p* < 0.001). Results were in the same direction with the HSCL-5, total score was significantly higher in women (mean = 1.88; SD 0.03) than in men (mean = 1.53; SD 0.02; *t* = 8.51; *p* < 0.001).

Table 4 shows the results of the CFA: the factor loading for each model and correlation in the two-factor models. All the factor loadings were positive, statistically significant (*p* < 0.001), and above 0.30. In fact, all factor loadings in the different versions were above 0.45. The range of loadings was 0.45–0.81 for the one factor HSCL-10, 0.50–0.83 for the one factor HSCL-5, 0.47–0.82 for the two correlated factor HSCL-10, and 0.51–0.85 for the two correlated factor HSCL-5. Item 17 “Feeling blue” was the item with the highest factor loadings in all the models analyzed. When analyzing the models with two correlated factors, a strong correlation between the two-factors of depression and anxiety was observed for both the HSCL-10 and the HSCL-5.

**TABLE 4:** Confirmatory factorial analysis: Factor loading values and correlation between two anxiety and depression factors.

	One factor		Two correlated factors			
	HSCL-10	HSCL-5	HSCL-10		HSCL-5	
			Anxiety	Depression	Anxiety	Depression
Item 1	0.45		0.49			
Item 2	0.52	0.50	0.56		0.54	
Item 3	0.60		0.62			
Item 4		0.64			0.71	
Item 7	0.66		0.69			
Item 12	0.57			0.58		
Item 16	0.62	0.63		0.64		0.64
Item 17	0.81	0.83		0.82		0.85
Item 20		0.58				0.57
Item 22	0.69			0.68		
Item 23	0.54			0.55		
Item 25	0.47			0.47		
Factor correlation			0.90		0.87	

The goodness-of-fit indices in the studied factor models can be consulted in Supplementary Table 1 of the Supplementary materials. Globally, the indices showed that the HSCL-10 and the HSCL-5 do not have a stable factor structure.

### Criterion validity and diagnostic accuracy: Relation of HSCL-10 and HSCL-5 with gold-standard CIDI

Of the 767 participants who completed the HSCL-25, 736 also took part in the CIDI interview (96.0%). Depression prevalence varied depending on the questionnaire employed (Table 5). Prevalence measured with the HSCL-10 was similar to that obtained with the full HSCL-25 version and higher than the value obtained with the HSCL-5.

**TABLE 5:** Prevalence of depression according to CIDI, HSCL-25, HSCL-10, and HSCL-5, total and by gender.

	Male (*n* = 341)	Female (*n* = 395)	Total (*n* = 736)
CIDI	4.7 (CI95% 2.7–7.5)	10.9 (CI95% 8.0–14.4)	8.0 (CI95% 6.2–10.2)
HSCL-25 (cutoff = 1.75)	16.7 (CI95% 12.9-21-1)	38.7 (CI95% 33.9–43.7)	28.5 (CI95% 25.3–31.9)
HSCL-10 (cutoff = 1.85)	12.6 (CI95% 9.3–16.6)	30.1 (CI95% 25.6–34.9)	22.0 (CI95% 19.1–25.2)
HSCL-5 (cutoff = 2.00)	19.4 (CI95% 15.3–24.0)	41.5 (CI95% 36.6–46.6)	31.3 (CI95% 27.9–34.7)

Table 6 shows sensitivity, specificity, positive, and negative predictive values for the total number of participants and by gender. Sensitivity was similar for both genders, especially in the HSCL-10, whilst specificity was better in men. Negative predictive values were >95% for both versions, these values were for the total population and by gender. All values were higher for the HSCL-10 than for the HSCL-5.

**TABLE 6:** Sensitivity, specificity, positive and negative predictive values, total and by gender in the HSCL-10, and the HSCL-5.

		Male (*n* = 341)			Female (*n* = 395)			Total (*n* = 736)		
		Index	CI 95% lower limit	CI 95% upper limit	Index	CI95% lower limit	CI 95% upper limit	Index	CI 95% lower limit	CI 95% upper limit
HSCL-10 cutoff point = 1.85	Sensitivity	81.3	57.0	93.4	79.1	64.8	88.6	79.7	67.7	88.0
	Specificity	90.8	87.1	93.5	75.9	71.1	80.0	83.0	80.0	85.7
	PPV	30.2	18.6	45.1	28.6	21.2	37.3	29.0	22.6	36.4
	NPV	99.0	97.1	99.7	96.7	93.9	98.3	97.9	96.4	98.8
HSCL-5 cutoff point = 2.00	Sensitivity	81.3	57.0	93.4	76.7	62.3	86.8	78.0	65.9	86.6
	Specificity	83.7	79.3	87.3	62.8	57.6	67.7	72.8	69.3	76.0
	PPV	19.7	11.9	30.8	20.1	14.7	26.9	20.0	15.3	25.6
	NPV	98.9	96.8	99.6	95.7	92.2	97.6	97.4	95.7	98.5

PPV, positive predictive value; NPV, negative predictive value.

### HSCL10 vs. CIDI

**FIGURE 1:**
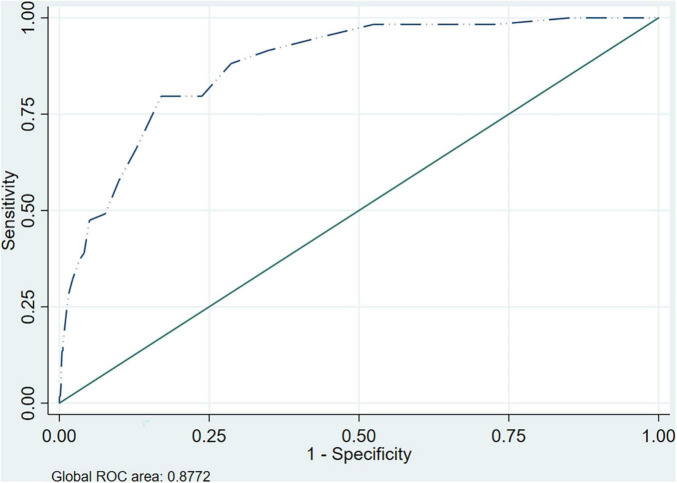
ROC curve and AUC HSCL-10 vs. CIDI.

The AUC between the HSCL-10 and the CIDI was 0.877 (CI95% 0.836–0.919). In the gender analysis it was greater in men with an AUC of 0.943 (CI95% 0.897–0.989) compared to women who had an AUC of 0.825 (CI95% 0.765–0.886). The ROC curve is depicted in Figure 1 and by gender in the Supplementary Figure 1.

### HSCL5 vs CIDI

**FIGURE 2:**
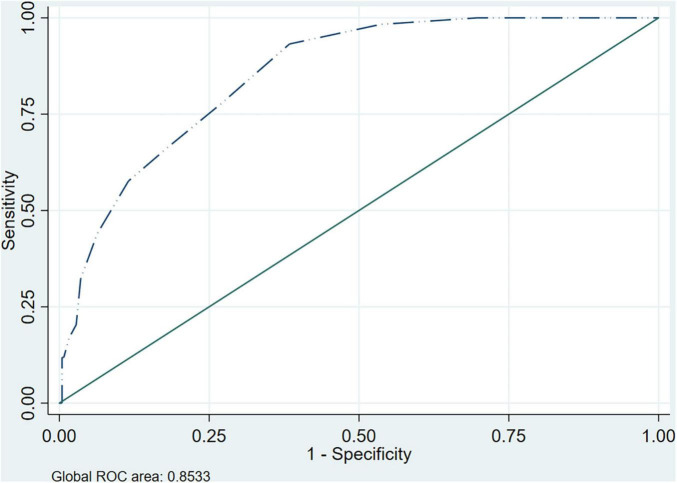
ROC curve and AUC HSCL-5 vs. CIDI.

The AUC between the HSCL-5 and the CIDI was 0.853 (CI95% 0.812–0.894). In the gender analysis it was greater in men with an AUC of 0.918 (CI95% 0.859–0.977) than in women who had an AUC of 0.795 (CI95% 0.734–0.855). The ROC curve is depicted in Figure 2 and by gender in the Supplementary Figure 2.

The optimum cutoff points for the study population were calculated with the Youden Index. For the HSCL-10 it was 1.90 for the total population and for both genders. For the HSCL-5 the optimum cutoff point was 1.80 for the total population and for women, and 2.00 for men. A table including sensitivity, specificity, positive, and negative predictive values using optimal cutoff points is available in the Supplementary Table 2.

## Discussion

This study is the first to analyze the psychometric properties and diagnostic accuracy of the Spanish versions of the HSCL-10 and HSCL-5, the results obtained now allow the use of both scales as screening tools for depression in the PC setting in Spain. The results demonstrated that in the Spanish PC population, the HSCL-10 presents high reliability and validity. The HSCL-5 also showed acceptable psychometric properties although slightly worse than the HSCL-10. Both scales showed adequate sensitivity and specificity when compared to the semistructured clinical interview CIDI conducted by trained professionals. The optimal cutoff points obtained were very close to those proposed by other authors.

The PC setting is ideal for the detection, diagnosis, and investigation of chronic, highly prevalent disorders as it allows an early study of such pathologies (48). With respect to depression, it is widespread in Spanish and European countries (40) yet patients frequently consult their FD for other motives than their mood disorders (6).

There are many questionnaires that can be employed as screening tools to detect depression at all its stages (49–52), some of which have been validated within the PC setting (1). Moreover, shortened versions have been proposed in order to provide a similar diagnostic value that saves time for both patient and healthcare professional (20, 53, 54). Recently, the HSCL-10 and HSCL-5 have shown acceptable reliability and validity (22, 27), particularly the HSCL-10 (25, 33, 47).

With respect to reliability, Cronbach’s Alpha results of 0.84 and 0.77 were obtained for the HSCL-10 and HSCL-5, respectively. As both were above 0.7, they are considered acceptable (55). Such findings are similar to those obtained by other authors (33) and, as occurred in other studies (19, 22, 27), the reliability of the two short versions was lower than the HSCL-25.

In the full 25-item version, 17 “Feeling blue” was the most consistent (18). This item which asks about sadness, a basic characteristic in patients with depression, is included in the HSCL-10 and the HSCL-5 and was also the most consistent in the two short versions. Once removed, reliability diminished to the minimum as can be seen in Table 3. The next most consistent items in the HSCL-25 were four “Nervousness” and seven “Feeling tense.” The former was included in the 5-item version and the latter in the 10-item one. Item 24 was the least consistent in the HSCL-25 and is not present in either of the short versions. The following least consistent items in the HSCL-25 were 8 “Headache” and 18 “Thinking of ending one’s life,” neither of which is included in the two short versions.

When analyzing Cronbach’s Alpha without the item in the short versions, it was observed that 25 “Sleep disturbance” worsened HSCL-10 reliability, that is to say, by eliminating this item reliability improved. Such a finding concurs with that reported by Kleppang et al. who employed the 10-item version with adolescents in Norway (25). The other items contributed to good reliability in both the HSCL-10 and HSCL-5.

Regarding analysis of the scale’s factorial structure, this was performed with the CFA as the HSCL-25 has been widely studied with one single factor or two correlated ones even though other models have been proposed (10). The HSCL-10 and the HSCL-5 maintain the same factor structure (27). The goodness-of-fit indices for the studied models were not optimal, showing that the data did not fit the hypothesized factor structure of one factor and two correlated factors. The factor structure was unstable for both scales, this is a limitation of our study. Further validation studies should be done to assess other alternative models of the factor structure of the HSCL-10 and the HSCL-5. By examining the factor loading of 10 and 5 items, respectively, all the items were significantly loaded to the hypothesized construct, and all factor loadings were statistically significant, positive, and above 0.45. Item 17 “Feeling blue” had the highest factor loadings in all models tested and for both the HSCL-10 and the HSCL-5. In the study of the two-factor models, there was a factorial correlation of 0.90 between the dimensions in the HSCL-10 and of 0.87 in the HSCL-5, these findings indicated that the depression and anxiety dimensions strongly correlated in a positive manner. The correlation is expectable as anxiety and depression are frequently found to be associated comorbidities (56).

The calculation of the total score is done in the same way in the different versions of the HSCL, by dividing the total score by the corresponding number of ítems answered. With respect to the total score, in comparison with the 25-item long version, the mean score of the HSCL-25 was 1.57, very similar to the 1.55 obtained with the HSCL-10. The mean score increased to 1.72 with the five-item version. The means were significantly greater in women for all three versions, this is unsurprising considering that depression is more prevalent in the female gender (40, 41). Significant differences reported in mean scores according to age and marital status for the 25 and 10-item versions were lost in the 5-item one.

With respect to prevalence, it was higher in the HSCL-5 followed by the HSCL-10 and then the HSCL-25. All three versions and the gold-standard CIDI showed greater prevalence in women than men. Employing the cutoffs corresponding to each version (22), sensitivity was similar for both genders whilst specificity was greater for men in the two short versions. Findings that concur with those reported for the HSCL-25 (18). In a study comparing the HSCL-10 with the CIDI (33), slightly higher results were reported with respect to sensitivity, although a different cutoff was employed. Another multicenter study conducted in General Practice in Norway and Denmark using the HSCL-10 and the CIDI obtained similar results to ours in terms of sensitivity and specificity (23).

Cutoffs can be based on previous studies, cutoffs used in clinical practice, cutoffs recommended by clinical practice guidelines, or cutoffs recommended by the original authors (57). There is very little literature on the appropriate cutoff point for the HSCL-10 and the HSCL-5, Strand et al. recommend 1.85 for the HSCL-10 and 2.00 for the HSCL-5 (22). By interpreting the results from ROC curves, the accuracy for various cutoffs was explored, and optimal cutoff values were obtained considering the maximum value of Youden Index. The optimum cutoff that we calculated for the study population is very close to that in the literature (22). Therefore, we considered that those of 1.85 and 2.00 for the HSCL-10 and HSCL-5, respectively, were appropriate to use showing adequate sensitivity, specificity, and AUC. The negative predictive values were >90% for the two short versions and both genders whilst the positive predictive values were low. Such a finding reinforces the need to complete the diagnosis of depression through a clinical interview.

Other authors have analyzed validity between the HSCL-25 and the two short versions with ROC curves (22). As we had the semi-structured CIDI interview for all our participants, we contrasted it against the two short versions with ROC curves. This is one of the strengths of the present study as sometimes there is a lack of gold-standard and the full version is used as a reference to assess validity. In other studies, only those participants who have a positive result on the scale and a small sample of those with a negative one, undergo the clinical interview of reference. The AUC was 0.88 for the HSCL-10 and 0.85 for the HSCL-5, both above the 0.75 cutoff considered to be of clinical utility, and greater than the 0.80 which confers a “good” classification (≥0.90 is considered “excellent”) in terms of discriminative properties of the diagnostic accuracy (58). A study carried out by Haavet et al. (33) also obtained an AUC of 0.88 when comparing the HSCL-10 with the CIDI, other studies with similar methodology have obtained a lower AUC (23). For both short versions the AUC was greater in men, thus the probability of accuracy in diagnosis in males is greater. Such a gender difference has also been reported for the longer HSCL-25 (18).

The main limitation of our study is that the psychometric properties of the two short versions were evaluated based on the responses to the 25-item scale as performed by other authors (22, 27). The study population came from the EIRA study (34, 35) and were patients aged 45–75 years presenting an unhealthy behavior. Adults often engage in two or more unhealthy behaviors simultaneously, the co-occurrence of unhealthy diet with insufficient physical activity ranges between 47 and 54%, unhealthy diet with smoking between 23 and 28%, and insufficient physical activity with smoking between 8 and 20% (59). Unhealthy diet can be associated to depression (60). Despite the limited age range and the selection criteria in the present study, the authors consider that the results are transferable given the large number of participants, moreover, they had attended the PHC for any motive, consequently, the sample is sufficiently representative.

When deciding which questionnaire to use, there are a few relevant factors to be taken into account, including the population involved and the setting. A balance must be achieved between psychometric properties and such pragmatic characteristics as self-administration, number of items, simplicity/interpretability of scores, and accessibility (61). Scales with a reduced number of items may be the best option as they are practical and feasible within the PC setting without excessively losing reliability and validity.

These findings indicate that the HSCL-10 and HSCL-5 questionnaires show adequate reliability and validity in order to be employed in PC to detect and evaluate depressive symptoms. With such a short number of items they are timesaving and facilitate the detection of cases of depression that could otherwise go unnoticed.

## Conclusion

The Spanish versions of the HSCL-10 and HSCL-5, especially the HSCL-10, are reliable and valid tools to detect depressive symptoms and can be used in Primary Care settings.

## Data availability statement

The raw data supporting the conclusions of this article will be made available by the authors, without undue reservation.

## Ethics statement

The studies involving human participants were reviewed and approved by the Clinical Research Ethics Committee of the University Institute Foundation for Research into Primary Health Care Jordi Gol from Barcelona (reference: P16/025). All the participants gave their written informed consent, and the trial was conducted in accordance with the ethical standards set out in the Declaration of Helsinki. Data were anonymously processed and were used only for the study objectives. Personal data confidentiality was ensured as set out in applicable regulations. Access to medical records was gained as indicated in the legislation currently in force. The patients/participants provided their written informed consent to participate in this study.

## Author contributions

MR-B, MF-S-M, AC, JL, and PN: conceptualization. EM and IG-G: data collection. MR-B, MF-S-M, and AC: formal analysis. MR-B, MF-S-M, and EP-R: writing—original draft preparation. All authors have reviewed and agreed to the published version of the manuscript.
