# Supplementary material for: Measuring depression in Primary Health Care in Spain: Psychometric properties and diagnostic accuracy of HSCL-5 and HSCL-10
Source: Front Med (Lausanne). 2023 Jan 9;9:1014340. doi: 10.3389/fmed.2022.1014340 (PMC9869680; doi:10.3389/fmed.2022.1014340)
Supplement: Supplementary file 1 [file Table_1.DOCX]

**SUPPLEMENTARY MATERIAL**

Table S1. Goodness-of-fit indices in studied factor models

| **Model** |  | **X^2^ (df)** | **p** | **CFI** | **TLI** | **RMSEA**  **(90% CI)** | **SRMR** |
| --- | --- | --- | --- | --- | --- | --- | --- |
| One factor | HSCL-10 | 291 (35) | <0.001 | 0.89 | 0.86 | 0.098  (0.087 - 0.108) | 0.056 |
|  | HSCL-5 | 53 (5) | <0.001 | 0.95 | 0.90 | 0.112  (0.086 – 0.140) | 0.041 |
| Two correlated factors | HSCL-10 | 269 (34) | <0.001 | 0.90 | 0.87 | 0.095  (0-084 - 0.206) | 0.054 |
|  | HSCL-5 | 42 (4) | <0.001 | 0.96 | 0.90 | 0.112  (0.083 – 0.144) | 0.039 |

X^2^: chi-square; df: degrees of freedom, CFI: Comparative Fit Index; TLI: Tucker-Lewis Fit Index; RMSEA: Root Mean Square Error of Approximation; SRMR: Standardized Root Mean Square Residual
