# Supplementary material for: Measuring depression in Primary Health Care in Spain: Psychometric properties and diagnostic accuracy of HSCL-5 and HSCL-10
Source: Front Med (Lausanne). 2023 Jan 9;9:1014340. doi: 10.3389/fmed.2022.1014340 (PMC9869680; doi:10.3389/fmed.2022.1014340)
Supplement: Supplementary file 2 [file Table_2.DOCX]

**SUPPLEMENTARY MATERIAL**

Table S2. Sensitivity, specificity, positive and negative predictive values, total and by gender in the HSCL-10 and the HSCL-5 using optimal cutoff points calculated by the Youden Index.

|  |  | **Male (n=341)** | | | **Female (n=395)** | | | **Total (n=736)** | | |
| --- | --- | --- | --- | --- | --- | --- | --- | --- | --- | --- |
|  |  | **Index** | **CI95% lower limit** | **CI95% upper limit** | **Index** | **CI95% lower limit** | **CI95% upper limit** | **Index** | **CI95% lower limit** | **CI95% upper limit** |
| **Optimal cutoff point** | | 1.90 | | | 1.90 | | | 1.90 | | |
| **HSCL-10** | **Sensitivity** | 81.3 | 57.0 | 93.4 | 79.1 | 64.8 | 88.6 | 79.7 | 67.7 | 88.0 |
|  | **Specificity** | 90.8 | 87.1 | 93.5 | 75.9 | 71.1 | 80.0 | 83.0 | 80.0 | 85.7 |
|  | **PPV** | 30.2 | 18.6 | 45.1 | 28.6 | 21.2 | 37.3 | 29.0 | 22.6 | 36.4 |
|  | **NPV** | 99.0 | 97.1 | 99.7 | 96.7 | 93.9 | 98.3 | 97.9 | 96.4 | 98.8 |
| **Optimal cutoff point** | | 2.00 | | | 1.80 | | | 1.80 | | |
| **HSCL-5** | **Sensitivity** | 81.3 | 57.0 | 93.4 | 95.3 | 84.5 | 98.7 | 93.2 | 83.8 | 97.3 |
|  | **Specificity** | 83.7 | 79.3 | 87.3 | 50.9 | 45.6 | 56.0 | 61.6 | 57.9 | 65.2 |
|  | **PPV** | 19.7 | 11.9 | 30.8 | 19.2 | 14.4 | 25.0 | 17.5 | 13.7 | 22.0 |
|  | **NPV** | 98.9 | 96.8 | 99.6 | 98.9 | 96.1 | 99.7 | 99.0 | 97.6 | 99.6 |

PPV: positive predictive value; NPV: negative predictive value.
