# Supplementary figures and images for: Measuring depression in Primary Health Care in Spain: Psychometric properties and diagnostic accuracy of HSCL-5 and HSCL-10
Source: Front Med (Lausanne). 2023 Jan 9;9:1014340. doi: 10.3389/fmed.2022.1014340 (PMC9869680; doi:10.3389/fmed.2022.1014340)

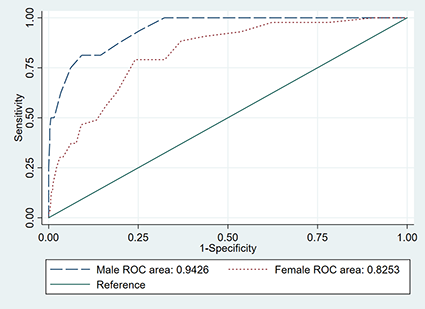

Supplement: Supplementary file 3 [file Image_1.TIF]

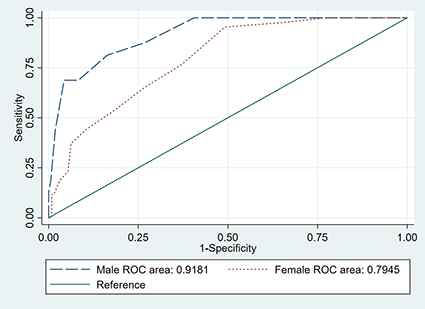

Supplement: Supplementary file 4 [file Image_2.TIF]
